# Objective analysis of perfluoropropane tamponade area after pars plana vitrectomy using ultra-widefield fundus stereographic projection images

**DOI:** 10.1038/s41598-020-75493-9

**Published:** 2020-10-26

**Authors:** Mihyun Choi, Suji Hong, Cheolmin Yun, Seong-Woo Kim

**Affiliations:** 1 Department of Ophthalmology, Korea University Guro Hospital, 148, Gurodong-ro, Guro-gu, Seoul, 08308 Republic of Korea; 2 Department of Ophthalmology, Korea University Ansan Hospital, 123, Jeokgeum-ro, Danwon-gu, Ansan-si, Gyeonggi-do Republic of Korea

**Keywords:** Retinal diseases, Outcomes research, Medical imaging

## Abstract

To objectively analyze the gas tamponade area in patients with different concentrations of perfluoropropane (C_3_F_8_) after pars plana vitrectomy (PPV), retrospective analysis was performed in patients diagnosed with retinal detachment or macular hole and underwent PPV with C_3_F_8_ tamponade of two concentrations (14% and 20%). The ultra-widefield fundus (UWF) images at one and 10 days and four weeks postoperatively were corrected using stereographic projection to adjust peripheral distortion. The gas–fluid interface curvatures were delineated using UWF stereographic projection images, and the gas–fluid area ratio and estimated gas area were calculated at each concentration. Among 65 eyes, 30 were in the 14% group and 35 were in the 20% group. The gas–fluid area ratio was 0.71 in the 14% group and 0.83 in the 20% group at 10 days (p = 0.046) and 0.27 and 0.45, respectively, at four weeks postoperatively (p < 0.001). The estimated gas area was 52.56 and 60.82 mm^2^ at 10 days (p = 0.025) and 19.83 and 33.86 mm^2^ at four weeks (p < 0.001). The gas tamponade areas were objectively shown to be greater under the 20% concentration than the 14% concentration of C3F8 at 10 days and 4 weeks postoperatively using UWF stereographic projection images.

## Introduction

Pars plana vitrectomy^1,2^ has been a procedure of choice in patients with retinal detachment^3^, macular hole^4^, and other vitreoretinal diseases^5^. And more than 50% of vitrectomies involve a gas tamponade to promote anatomical retinal re-attachment or macular hole closure^2^. With the advantages of high surface tension and a wide contact angle^6^, gases such as sulphahexafluoride, hexafluoroethane, or perfluoropropane (C_3_F_8_) are commonly used mixed with air during vitrectomy^7^. To attain a successful tamponade effect on retinal breaks including sufficient area and duration with enough surface tension and buoyancy^8^, a gas’s expansivity and longevity in the eye are important.

Though the term “nonexpansion concentration” is used, all gases expand after they are inserted into the vitreous due to the transfer of blood gases into the bubbles. Subsequently, the partial pressures of nitrogen, oxygen, and carbon dioxide with the retinal blood gas partial pressures are equalized and absorbed slowly^9,10^. For C_3_F_8_, 10% to 17% concentrations have been suggested as possible concentrations to prevent excessive expansion and the elevation of intraocular pressure (IOP)^11–13^. Previously, many studies were conducted on gas behavior and longevity in the eye, but most experiments were completed using animal models^14–16^ or predicted through experimental models^17,18^, and there is a shortage of reports that objectively analyzed changes in intravitreal gas in the human eye^19,20^. Still, many surgeons choose gas concentration based on subjective preference. According to a survey among surgeons using C_3_F_8_, the concentration choice varied from 12 to 20%^12^.

Ultra-widefield (UWF) fundus imaging, which can obtain wide-angle fundus images of about 180°–200° covering the peripheral retina, became the standard-of-care for diagnosis and screening of retinal disease^21,22^. Most observations of gas behavior in humans have studied estimated gas volume through the gas-height level seen through the dilated pupil^23^ or using A-scan ultrasound^20^. Unlike those studies, widefield fundus imaging which have large depth of focus allows the peripheral retina, intraocular gas bubble to be in focus simultaneously in gas-filled eyes and has made it possible to measure the gas–fluid level objectively. The UWF images digitally project the three-dimensional retina onto a two-dimensional image, resulting in distortion of the peripheral image that results in inaccurate measurement. A recent technique of stereographical projection of all relevant pixels to a plane through the equator of the eye provided relatively accurate measurements of the area from the posterior pole to the retinal periphery^24–26^.

The aim of this study was to objectively visualize the gas–fluid interface after perfluoropropane tamponade and to estimate the longevity in two different percentages of perfluoropropane gas presumed to be nonexpansile (14%) and slightly expansile (20%) after pars plana vitrectomy using stereographical projection images. Effect of gas concentration on intraocular pressure was also evaluated.

## Results

Among 237 patients who underwent vitrectomy with gas tamponade during the study period, 65 cases that satisfied the inclusion criteria were analyzed. Of them, 30 eyes were injected with 14% C_3_F_8_ and 35 eyes were injected with 20% C_3_F_8_ after vitrectomy. The patient demographics for the two gas concentration groups are described in detail in Table 1. A difference was not identified in comparison in age, sex, laterality, surgery indications, scleral suture, and preoperative best-corrected visual acuity (BCVA) and IOPs according to gas concentration.

**Table 1:** Patient demographics.

	14% (n = 30)	20% (n = 35)	p-value
Mean age (SD), years	63.10 (8.06)	59.00 (11.34)	0.103
Sex (M/F)	13/17	19/16	0.379
OD/OS	21/9	23/12	0.831
Axial length (SD), mm	23.74 (1.05)	23.77 (0.96)	0.902
Pre-op LogMAR BCVA, mean (SD)	0.99(0.77)	0.84 (0.74)	0.399
IOP at preoperation, mean (SD), mmHg	14.66 (3.68)	14.85 (3.41)	0.638
Scleral suture (%), n	18 (60)	20 (57)	0.492
**Diagnosis**			
RRD (%), n	17 (57)	27 (77)	0.187
MH (%), n	13 (43)	8 (23)	

Data are presented as N (%) or mean (SD).

*SD* standard deviation, *BCVA* best-corrected visual acuity, *IOP* intraocular pressure, *RRD* rhegmatogenous retinal detachment, *MH* macular hole.

### Gas fluid interface

**Figure 1:**
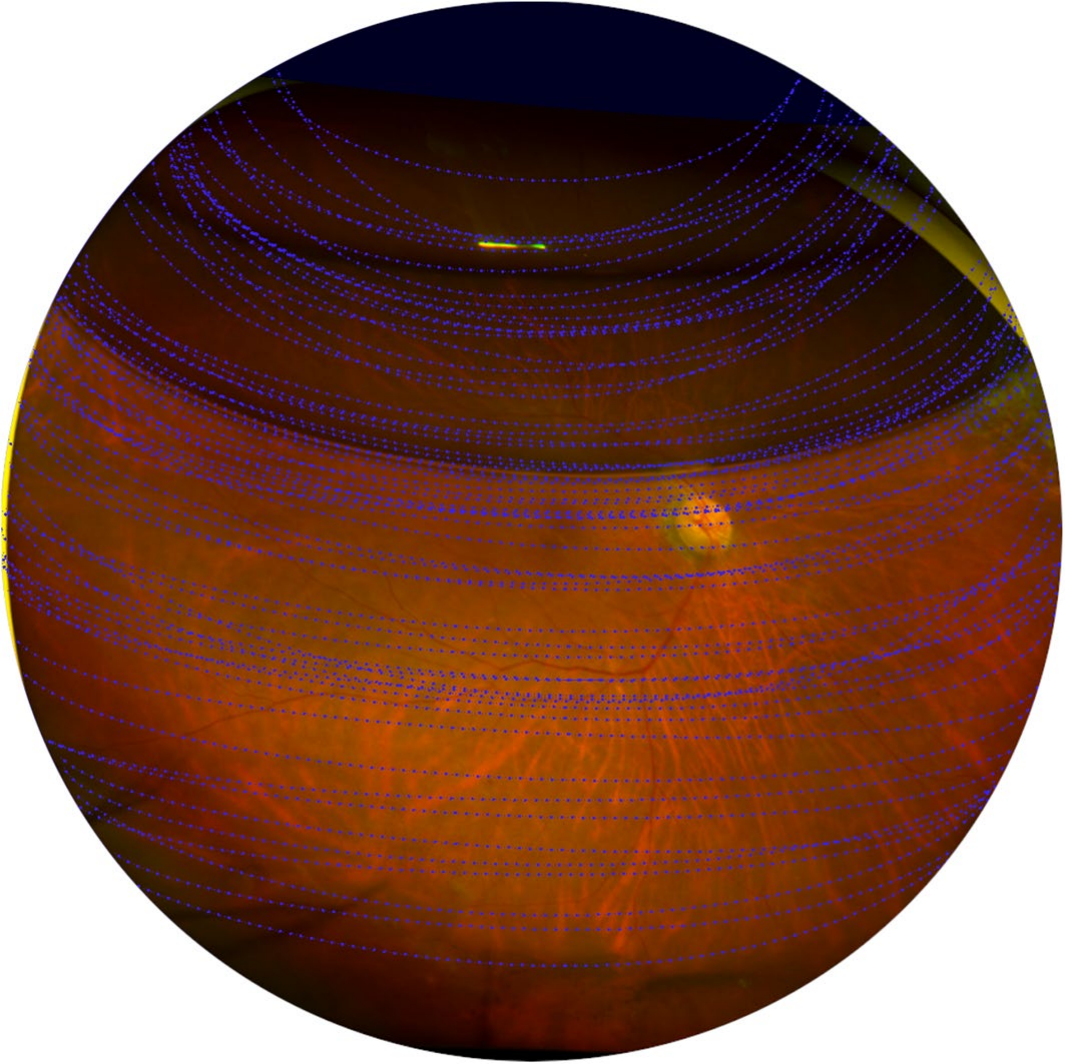
Synthesis of all gas–fluid curvatures in a UWF stereographic projection image. Using the projection image, the distortion of the periphery was reduced and the change in the gas–fluid interface was objectively confirmed.

In the UWF stereographic projection images of patients taken at 10 days and four weeks after surgery, gas–fluid curvature was collected and synthesized in a projection retinal image to directly and intuitively report the change in gas–fluid interface in the eye (Fig. 1). The boundary between the gas and the fluid appeared relatively flat in the lower hemisphere but, as gas was absorbed, the boundary showed a curved meniscus shape, while, as the volume of gas in the eye decreased, the boundary became curved with a greater spherical shape.

### Gas tamponade area

The estimated gas area in the virtual coronal plane of the eyeball using a UWF stereographic projection image compared between each gas concentration is summarized in Table 2. The proportion of gas area in stereographic projection retinal image at 10 days after surgery was 0.71 ± 0.09 in the 14% group and 0.83 ± 0.07 in the 20% group (p = 0.046). At 4 weeks postoperatively, it was 0.27 ± 0.15 and 0.45 ± 0.15, respectively (p < 0.001). The estimated area of gas in virtual coronal plane image of eyeball was 52.56 ± 7.27 mm^2^ in the 14% group and 60.82 ± 4.64 mm^2^ in the 20% group at 10 days postoperatively (p = 0.025) and 19.83 ± 11.55 mm^2^ and 33.86 ± 11.00 mm^2^, respectively at 4 weeks postoperatively (p < 0.001).

**Table 2:** Estimated gas area in the virtual coronal plane of an eyeball using UWF stereographic projection Images.

Postoperative day	14% (n = 30)		20% (n = 35)		p-value	
	Ratio	Estimated area (mm^2^)	Ratio	Estimated area (mm^2^)	Ratio	Estimated area (mm^2^)
10 days (± 1)	0.71 (0.09)	52.56 (7.27)	0.83 (0.07)	60.82 (4.64)	**0.046**	**0.025**
28 days (± 3)	0.27 (0.15)	19.83 (11.55)	0.45 (0.15)	33.86 (11.00)	** < 0.001**	** < 0.001**

Data are presented as mean (standard deviation).

Bold values indicate statistically significant of p < 0.05 level.

### Postoperative IOP changes

IOP at one, three, and 10 days and 1 and 2 months postoperatively was 16.37 ± 5.61 (minimum–maximum: 7–31), 16.62 ± 5.52 (9–33), 16.37 ± 4.63 (10–28), 15.38 ± 3.10 (10–27), and 15.04 ± 3.56 (11–24) mmHg in the 14% group and 17.63 ± 6.14 (8–33), 16.43 ± 6.91 (7–38), 15.27 ± 4.30 (6–27), 15.23 ± 3.29 (8–21), and 14.27 ± 2.77 (8–19) in the 20% group. The effect of gas concentration on postoperative IOP was not statistically significant (p = 0.559, repeated-measures analysis of variance test). Relative to preoperative IOP, the IOP was increased significantly on the first day after vitrectomy in the 20% group (p = 0.016), which showed no difference at three days (p = 0.167), 10 days (p = 0.383), 1 month (p = 0.472), and two months (p = 0.377) postoperatively. In the 14% group, IOPs at 1 day (p = 0.627), three days (p = 0.095), 10 days (p = 0.122), one month (p = 0.262), and 2 months (p = 0.562) after surgery were not statistically different from the preoperative IOP. There was no significant difference in IOP on the first day after surgery when compared between the two groups (p = 0.143). The number of eyes with ocular hypertension at any time during the follow-up period was 10 eyes (33.3%) in the 14% group and 14 eyes (31.1%) in the 20% group (p = 0.800). Ocular hypertension was seen in four eyes on the first day, three eyes on the third day, and three eyes on the 10th day postoperatively in the 14% group but nine eyes on the first day and five eyes on the third day in the 20% group (p = 0.087). The total number of anti-glaucoma eyedrops added during the postoperative follow-up period was 0.63 ± 0.85 in the 14% group and 0.45 ± 0.64 in the 20% group (p = 0.346). One eye in the 14% group showed peripheral anterior synechiae at 2 months after surgery, for which she was referred to a glaucoma specialist and underwent laser iridotomy.

## Discussion

Through this study, we were able to visualize the shape of the gas tamponade in the vitreous cavity that was mathematically presented by Eames et al.^17^ and Bahill^27^. The curvature was a relatively flat horizontal surface in the lower hemisphere and changed to a meniscus or lens shape in the upper hemisphere. This not only supports the existence of a good match with previous mathematical models but also has the advantage of being clinically useful for gas shape prediction. Due to spherical shape properties, the contact arc changes rapidly at the inferior and superior positions and, when the gas volume in the vitreous cavity decreases below 50%, the contact surface sharply decreases^28^, so it would not have enough force to cover other retinal breaks that are not located in the superior area or to maintain gas-fovea contact after surgery for macular hole. In particular, as shown in Fig. 1, when the gas area is less than 50%, the gas–fluid interface has a more convex meniscus curve, and the contact arc is reduced more rapidly.

One of the concerns of surgeons when injecting gas after vitrectomy is the ideal concentration that can fill the vitreous cavity as much as possible without causing increased IOP. The retinal adhesive force through laser retinopexy stabilizes after 2 to 3 weeks and the cryotherapy weakens the adhesive force due to local inflammation in the first week after surgery^29,30^. In other words, to induce retina re-attachment, it is necessary to apply a force for a sufficient period of at least 2 to 3 weeks. In vitrectomy for macular hole, gas-fovea contact is essential for hole closure, and a long duration of intraocular gas tamponade showed a higher success rate for macular hole surgery (in comparing 16% vs. 10% C_3_F_8_)^31,32^. Although non-inferiority of shorter-acting gas has been reported in recent studies^33^, there is still a surgeon preference for long acting gas^4,34^. In both indications, gas is injected to completely fill the vitreous cavity and achieve a larger gas-retinal contact area. But this is seldom achieved because of residual subretinal or preretinal fluid or fluid in the anterior vitreous area. For this reason, Williamson et al. suggested the theoretical gas concentration necessary to achieve 100% fill of the vitreous cavity is 13% to 19%, when assuming 20% to 30% vitreous fluid remains after fluid–gas exchange^9^. Tamponade of slightly expansile gas is based on this theoretical calculation.

Previously, Han et al. compared the effects of 12% and 20% C_3_F_8_ gas on longevity and IOP in 30 patients and reported that the intraocular longevity was 6.7 weeks for 12% C_3_F_8_ and 8.4 weeks for 20% C_3_F_8_, with no significant effect on IOP elevation seen between the two different concentrations ^23^. In this study, the gas area occupied 83% of the virtual coronal plane of the eye in the 20% group at 10 days after surgery and 45% at 4 weeks after surgery. As C_3_F_8_ is known to produce the maximal expansion on the third day and absorbed thereafter^11^, this suggests that 20% C_3_F_8_ nearly filled the vitreous cavity at early postoperative days and remained as a larger tamponade area at 1 month postoperatively to achieve anatomical success. It is reported that the decline in the volume of the gas bubble follows a first-order exponential decay, showing bubble to have a constant “half-life” and be independent of the injected volume^15,28^. “Half-life” shows a linear increase with gas concentration^13^, which can help explain the larger tamponade area in the 20% C_3_F_8_ in our study.

The use of expansile concentrations has been reported as a risk for IOP elevation^35^. However, in this study, there was an increase in IOP relative to the preoperative state in the 20% group on the first day after surgery (p = 0.016) but with no statistical difference relative to that in the 14% group (p = 0.143). Perhaps adequate outflow facility in these non-glaucomatous, pseudophakic eyes may have accommodated slow expansion without excessive pressure elevation. In the 20% group, ocular hypertension was often observed on the first day after surgery and, because the anti-glaucomatous eyedrop was applied earlier, the effect on the IOP of 20% C_3_F_8_ might be masked. In other words, it is suggested that IOP elevation after fluid–gas exchange with 20% C_3_F_8_ gas can be adjusted with a proper anti-glaucomatous eyedrop. These findings also support that IOP monitoring should be performed closely, regardless of the concentration used, as there was a patient with an increase in IOP at both concentrations.

There are some limitations in this study. This was a retrospective study of two indications that could not be compared with the success rate of surgery according to concentration. Both the mixed indications and the retrospective design prevent determination of the optimal concentration through this study. Further research is necessary to compare the surgical success rate within a single indication to find clinical benefit for longer duration of gas tamponade. In addition, the gas concentrations were chosen according to the preference of the operator and the timing of surgery between the two groups was different. It is thought that the difference in the timing of the operation did not affect the main outcome, but this should be considered in interpreting the results. Further, it was not possible to compare the longevity by comparing the fundus image until the gas had been completely absorbed. However, considering that the gas tamponade area at 4 weeks postoperatively was significantly larger in 20% C_3_F_8_ than 14%, the longevity could also be estimated to be longer than 14% for 20% C3F8. In conclusion, we have demonstrated the UWF stereographic projection image as a useful methods for evaluation of gas tamponade state and we found that 20% C_3_F_8_ in vitrectomy and fluid–gas exchange has advantages over 14% C_3_F_8_ in terms of its sufficient retinal lesion cover. In nonglaucomatous, pseudophakic or aphakic eyes, the surgeon can choose the gas concentration by closely monitoring the IOP.

## Material and methods

A nonrandomized, consecutive case series and retrospective analysis were performed on cases undergoing pars plana vitrectomy with gas tamponade at the Korea University Guro Hospital and Korea University Ansan Hospital between January 2016 and October 2019. The Institutional Review Board (IRB) of Korea University Medical Center approved this study (IRB no. 2020GR0147) and all research and data collection processes were conducted in accordance with the tenets of the Declaration of Helsinki. The Institutional Review Board of Korea University Medical Center waived the need for written informed consent from the participants, because of the study’s retrospective design.

### Patient selection

Patients within 20 to 80 years of age and diagnosed retinal detachment and macular hole were included. Patients who had previously undergone scleral buckle or encircle surgery, high myopia with an axial length of more than 25 mm, history of glaucoma or ocular hypertension and history of trauma were excluded. Only pseudophakic eyes were included, and cases of posterior capsular rupture or zonulysis during phacoemulsification were excluded as gas absorption could be affected by lens status and zonular instability^13^.

### Data sources

All patients received a complete examination before vitrectomy: slit lamp examination, fundus examination, BCVA, measurement of IOP, UWF fundus imaging (Optos Inc., Dunfermline, UK), axial length measurement using the IOL Master 500 (Carl Zeiss Meditec AG, Jena, Germany).

### Operation

All patients underwent three-port, 23-gauge pars plana vitrectomy [D.O.R.C. Associate; Dutch Ophthalmic Center (International) BV, Zuidland, the Netherlands] conducted by a single vitreoretinal surgeon (S. W. K.) with the same surgical techniques during the study period. Three ports were prepared by inserting trocar cannulas into the sclera 3 mm from the limbus at the inferotemporal, superotemporal and superonasal sides. An infusion line which continuously supplied balanced salt solution (BSS; Alcon, Fort Worth, TX) was connected to the inferotemporal port. Total vitrectomy with posterior vitreous detachment was performed with an indirect lens (Oculus Biom 5, Oculus Surgical, Inc., FL, USA) in all patients. During the surgery, the peripheral vitreous and anterior vitreous cortex were removed as much as possible by staining the remnant cortex with a triamcinolone acetonide injection (Maqaid, Hanmi Pharm.Co.,Ltd., Seoul, Korea). At the last step of the operation, fluid–air exchange was performed through the infusion cannula using the vented-gas forced-infusion (VGFI; Associate; DORC, Zuidland, Netherlands) and a soft tip instrument (backflush needle) as much as possible by repeatedly waiting and removing the fluid over the posterior pole of the retina. A 50-cc syringe of C_3_F_8_ gas (Teknogases ready-to-use gas; Teknomek Medical, Istanbul, Turkey) diluted with air as 14% and 20% connected to the infusion cannula and opposite port from infusion cannula was opened. The surgeon used only C_3_F_8_ in vitrectomy cases which needed gas tamponade and used a 14% concentration of C_3_F_8_ from January 2016 to February 2018 and switched to a 20% concentration of C_3_F_8_ from March 2018 to October 2019 because the surgeon’s preference had changed. A sufficient amount of diluted gas was injected over 40 cc while keeping the other port opened such that the vitreous cavity was fully filled with gas. After the cannula was extruded from the eye, if a bubble was observed at the sclerotomy site, the sclera was sutured.

### Follow up

**Figure 2:**
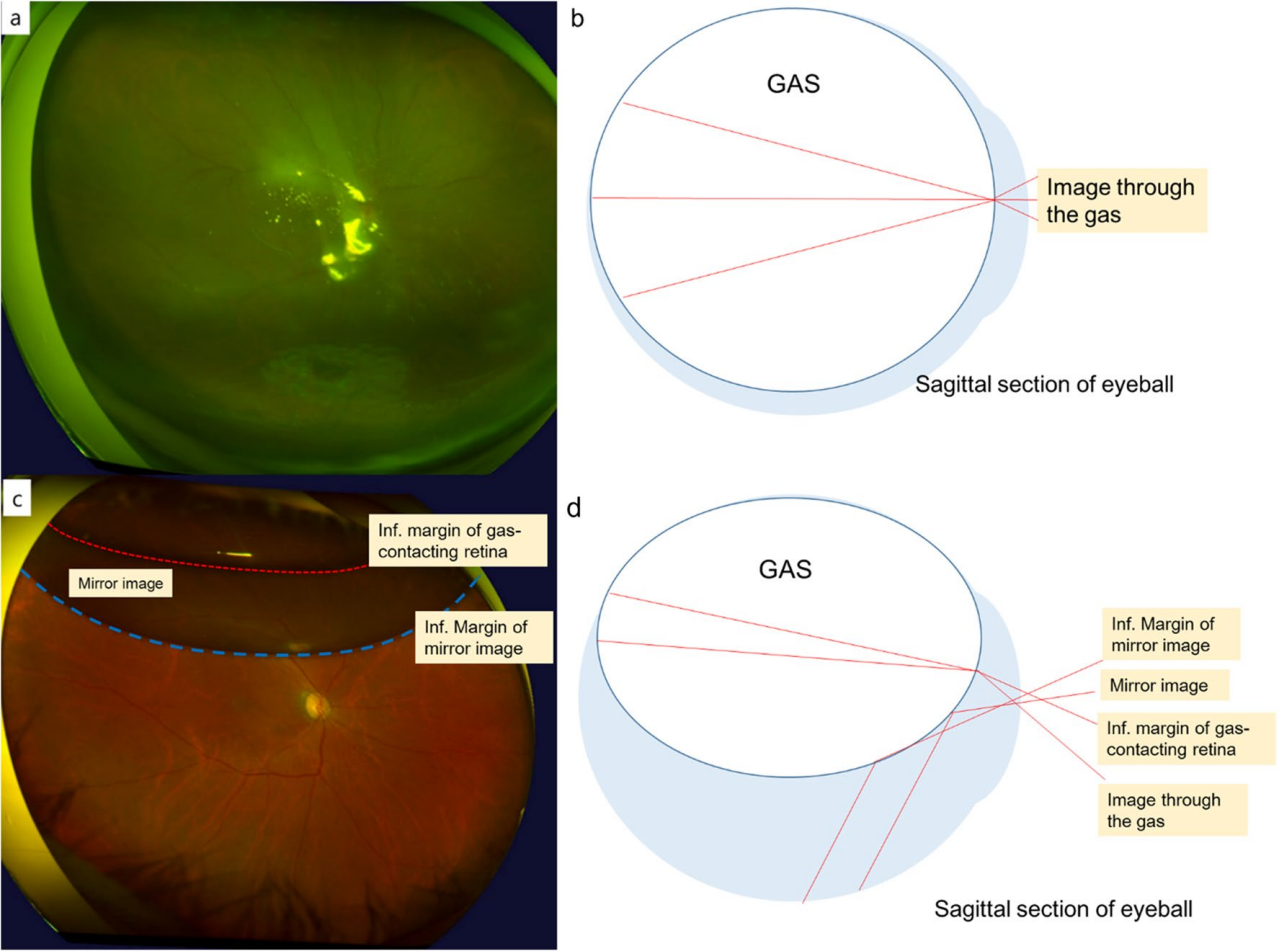
UWF fundus images and schema of an eye with gas tamponade**.** When the gas filled below the dilated pupil margin, the gas–fluid margin was not found **(a,b)**. Only those patients who showed no gas–fluid margin at one day postoperatively were included in this analysis. As the gas was absorbed, the gas–fluid curvature presented as a banana shape **(c,d)**. The superior oval area originates from the image seen thorough the gas bubble and the inferior banana-shaped area from the reflecting mirror image of the inferior retina. The upper margin of the mirror image (red dotted line) was thought to be the gas boundary that contacted the retina. The lower margin of the mirror image from the reflection of the gas surface (blue dotted line) was thought to be the inferior margin of the gas.

Follow-up visits were performed at one, three, and 10 days and one and two months postoperatively. At every visit, all patients underwent IOP measurement, slit lamp examination and dilated fundus examination. UWF fundus images were taken at one and 10 days (± 1 days) and 4 weeks (± 3 days) postoperatively after pupillary mydriasis. Patients without UWF images and patients whose gas–fluid interfaces were observed during UWF imaging at one day after surgery were excluded as from consideration due to insufficient gas tamponade in surgery or postoperative leak of gas. When the amount of gas in the vitreous cavity was sufficient so that the boundary was not visible in the dilated pupil (more than 80% according to the classical definition^19^), no gas–fluid interface was observed on the UWF image (Fig. 2a,b). Anti-glaucoma eyedrops were added in patients with an IOP elevation of 21 mmHg or more. Patients with sustained ocular hypertension (IOP > 21 mmHg) who were not controlled were referred to a glaucoma specialist.

### Gas tamponade area measurement

**Figure 3:**
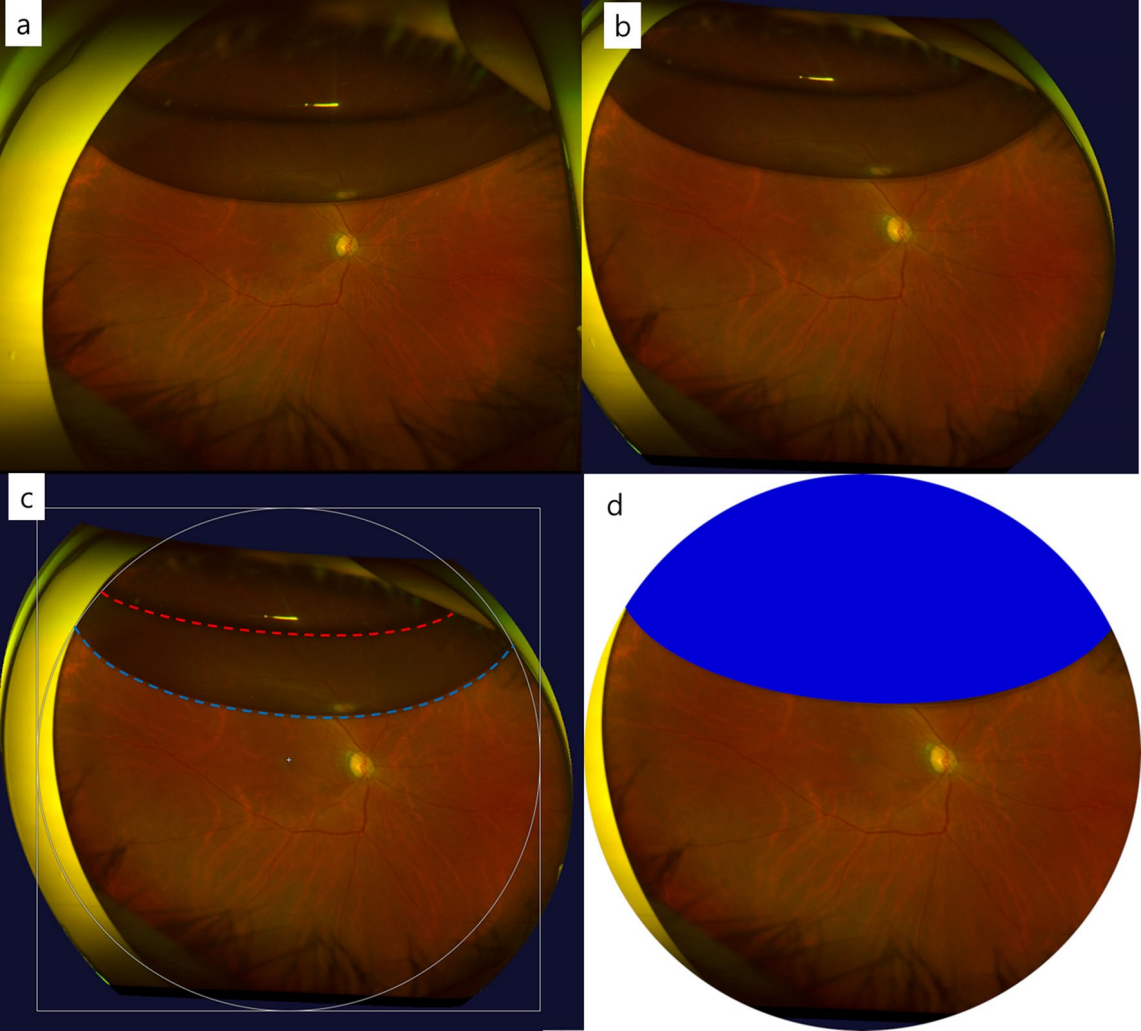
Image processing for the measurement of the gas-tamponade area. **(a)** A conventional UWF fundus image. **(b)** A UWF stereographic projection image from the reconstruction of A. Note that the gas–fluid margin shows a much steeper curvature from the mid-periphery to the far-periphery relative to the conventional UWF image. **(c)** A circle was drawn to contain the gas–fluid curvature centered on the macula and set as a virtual coronal plane. The upper margin of the mirror image (red dotted line) shows the gas boundary that contacted the retina and the lower margin of the mirror image (blue dotted line) shows the inferior margin of the gas. **(d)** The gas tamponade area (blue painted) in the virtual coronal plane is measured in this circle as a ratio.

To avoid peripheral image distortion, UWF stereographical projection images (three-dimensional wrap eye model images) were analyzed using software available from the manufacturer (V2 Vantage Pro version 2.11; Optos Inc., Dunfermline, UK). There was a significant difference in the gas–fluid curvature between the conventional UWF image and stereographical projection image, especially when the gas was less than 50% in the eye (Fig. 3a,b). After the gas was absorbed partially, the image of the retina through the gas (upper ovoid area) and the reflected image of the inferior retina from the gas surface (lower banana shaped area) were observed (Fig. 2c,d). We considered the inferior line of the reflected image as the gas–fluid interface (boundary)^22^. To reduce underestimation of the gas in the vitreous cavity in the UWF stereographical projection images due to artifacts (e.g., patient's eyelids and eyelash), we established a circle centered on the macula that contained both ends of the horizontal gas-fluid interface as the horizontal angle was usually larger than the vertical angle of field due to the patient’s eyelid using the open-source GNU Image Manipulation Program (GIMP 2.8.14) (Fig. 3c). Based on the fact that wide-field fundus photography has an angle of about 180° (reported as 180° to 200°), we adopted a circle as the “virtual coronal eyeball plane” that intersected the equator of the eye, with the fovea and cornea as poles^26^. The gas–fluid curvature was delineated using a Bézier curve model (Fig. 3c, blue dotted line), and gas–fluid curvatures from each UWF image were collected and synthesized (Fig. 1). Though the actual eyeball is not a perfect sphere, it is modeled as such, and the virtual coronal plane of the eye was assumed to be a circle whose diameter was the axial length of the patient’s eyeball. For this assumption, we excluded myopic eyes (axial length > 25 mm) which might have a more aspherical shaped eyeball contour. Based on this assumption, the diameter of the “virtual coronal plane” was defined as the axial length of the patient’s eyeball and the area was calculated. The gas tamponade area in projection image was measured using ImageJ software (National Institutes of Health, Bethesda, MD, USA) as a pixel ratio (Fig. 3d) and calculated to estimate the gas area in the “virtual coronal plane” using axial length.

### Statistical analysis

For statistical analysis, SPSS (SPSS version 20.0 for Windows; IBM Corp., Armonk, NY, USA) was used. BCVA was converted to the logarithm of the minimal angle of resolution (logMAR) and analyzed. The independent sample t-test used to compare continuous variables and the chi-squared test were used in comparing for categorical variables. As IOP were repeatedly measured from the same subject over time, a repeated-measures analysis of variance (ANOVA) test was used to compare two groups (14% vs. 20% C_3_F_8_).

## Data Availability

The raw data for this study is available upon reasonable request from the corresponding author.

## References

[1] McLaughlin MD, Hwang JC (2017). Trends in vitreoretinal procedures for medicare beneficiaries, 2000 to 2014. Ophthalmology.

[2] Gupta B, Neffendorf JE, Williamson TH (2018). Trends and emerging patterns of practice in vitreoretinal surgery. Acta Ophthalmol..

[3] Reeves MG, Pershing S, Afshar AR (2018). Choice of primary rhegmatogenous retinal detachment repair method in US commercially insured and medicare advantage patients, 2003–2016. Am. J. Ophthalmol..

[4] Elhusseiny AM, Schwartz SG, Flynn HW, Smiddy WE (2020). Long-term outcomes after macular hole surgery. Ophthalmol. Retina.

[5] Huang CH, Hsieh YT, Yang CM (2017). Vitrectomy for complications of proliferative diabetic retinopathy in young adults: Clinical features and surgical outcomes. Graefes Arch. Clin. Exp. Ophthalmol..

[6] Fawcett IM, Williams RL, Wong D (1994). Contact angles of substances used for internal tamponade in retinal detachment surgery. Graefes Arch. Clin. Exp. Ophthalmol..

[7] Chang S, Lincoff HA, Coleman DJ, Fuchs W, Farber ME (1985). Perfluorocarbon gases in vitreous surgery. Ophthalmology.

[8] de Juan, E., Jr., McCuen, B. & Tiedeman, J. Intraocular tamponade and surface tension. *Surv. Ophthalmol.* **30,** 47–51, 10.1016/0039-6257(85)90088-8 (1985). 10.1016/0039-6257(85)90088-8 4035558

[9] Williamson TH, Guillemaut JY, Hall SK, Hutter JC, Goddard T (2018). Theoretical gas concentrations achieving 100% fill of the vitreous cavity in the postoperative period: A gas eye model study. Retina.

[10] Hutter J, Luu H, Schroeder L (2002). A biological model of tamponade gases following pneumatic retinopexy. Curr. Eye Res..

[11] Peters MA, Abrams GW, Hamilton LH, Burke JM, Schrieber TM (1985). The nonexpansile, equilibrated concentration of perfluoropropane gas in the eye. Am. J. Ophthalmol..

[12] Kontos A, Tee J, Stuart A, Shalchi Z, Williamson TH (2017). Duration of intraocular gases following vitreoretinal surgery. Graefes Arch. Clin. Exp. Ophthalmol..

[13] Thompson, J. T. Kinetics of intraocular gases. Disappearance of air, sulfur hexafluoride, and perfluoropropane after pars plana vitrectomy. *Arch. Ophthalmol.* **107,** 687–691, 10.1001/archopht.1989.01070010705031 (1989). 10.1001/archopht.1989.01070010705031 2719578

[14] Lincoff H, Stergiu P, Smith R, Movshovich A (1992). Longevity of expanding gases in vitrectomized eyes. Retina.

[15] Lincoff H, Maisel JM, Lincoff A (1984). Intravitreal disappearance rates of four perfluorocarbon gases. Arch. Ophthalmol..

[16] Lincoff H (1980). Intravitreal longevity of three perfluorocarbon gases. Arch. Ophthalmol..

[17] Eames I, Angunawela RI, Aylward GW, Azarbadegan A (2010). A theoretical model for predicting interfacial relationships of retinal tamponades. Invest. Ophthalmol. Vis. Sci..

[18] Parver LM, Lincoff H (1978). Mechanics of intraocular gas. Invest. Ophthalmol. Vis. Sci..

[19] Wong RF, Thompson JT (1988). Prediction of the kinetics of disappearance of sulfur hexafluoride and perfluoropropane intraocular gas bubbles. Ophthalmology.

[20] Jacobs PM, Twomey JM, Leaver PK (1988). Behaviour of intraocular gases. Eye (Lond).

[21] Nagiel A, Lalane RA, Sadda SR, Schwartz SD (2016). Ultra-widefield fundus imaging: A review of clinical applications and future trends. Retina.

[22] Inoue M, Koto T, Hirota K, Hirakata A (2017). Ultra-widefield fundus imaging in gas-filled eyes after vitrectomy. BMC Ophthalmol..

[23] Han, D. P., Abrams, G. W., Bennett, S. R. & Williams, D. F. Perfluoropropane 12% versus 20%. Effect on intraocular pressure and gas tamponade after pars plana vitrectomy. *Retina* **13,** 302–306 (1993). 8115730

[24] Croft DE (2014). Precise montaging and metric quantification of retinal surface area from ultra-widefield fundus photography and fluorescein angiography. Ophthal. Surg. Lasers Imaging Retina.

[25] Sagong, M., van Hemert, J., Olmos de Koo, L. C., Barnett, C. & Sadda, S. R. Assessment of accuracy and precision of quantification of ultra-widefield images. *Ophthalmology* **122,** 864–866, 10.1016/j.ophtha.2014.11.016 (2015). 10.1016/j.ophtha.2014.11.016 25576995

[26] Tan CS (2016). Measuring the precise area of peripheral retinal non-perfusion using ultra-widefield imaging and its correlation with the ischaemic index. Br. J. Ophthalmol..

[27] Bahill AT (2016). Model for absorption of perfluoropropane intraocular gas after retinal surgeries. Int. J. Med. Health Sci. Res..

[28] Neffendorf JE, Gupta B, Williamson TH (2018). The role of intraocular gas tamponade in rhegmatogenous retinal detachment: A synthesis of the literature. Retina.

[29] Yoon YH, Marmor MF (1988). Rapid enhancement of retinal adhesion by laser photocoagulation. Ophthalmology.

[30] Kita M, Negi A, Kawano S, Honda Y (1991). Photothermal, cryogenic, and diathermic effects of retinal adhesive force in vivo. Retina.

[31] Thompson JT, Glaser BM, Sjaarda RN, Murphy RP, Hanham A (1994). Effects of intraocular bubble duration in the treatment of macular holes by vitrectomy and transforming growth factor-beta 2. Ophthalmology.

[32] Thompson JT, Smiddy WE, Glaser BM, Sjaarda RN, Flynn HW (1996). Intraocular tamponade duration and success of macular hole surgery. Retina.

[33] Essex RW (2016). The effect of postoperative face-down positioning and of long- versus short-acting gas in macular hole surgery: Results of a registry-based study. Ophthalmology.

[34] Sigler EJ, Randolph JC, Charles S, Calzada JI (2012). Intravitreal fluorinated gas preference and occurrence of rare ischemic postoperative complications after pars plana vitrectomy: A survey of the American Society of Retina Specialists. J. Ophthalmol..

[35] Chen PP, Thompson JT (1997). Risk factors for elevated intraocular pressure after the use of intraocular gases in vitreoretinal surgery. Ophthal. Surg. Lasers.

